# An Epigenetic Insight into NLRP3 Inflammasome Activation in Inflammation-Related Processes

**DOI:** 10.3390/biomedicines9111614

**Published:** 2021-11-04

**Authors:** Aroa Baragaño Raneros, Cristian Ruiz Bernet, Aida Bernardo Flórez, Beatriz Suarez-Alvarez

**Affiliations:** Translational Immunology Laboratory, Instituto de Investigación Biosanitaria del Principado de Asturias (ISPA), Hospital Universitario Central de Asturias (HUCA), 33011 Oviedo, Spain; baraganoaroa@hotmail.com (A.B.R.); cristian.ruiz.bernet@gmail.com (C.R.B.); aidabernardoflorez@gmail.com (A.B.F.)

**Keywords:** NLRP3, epigenetics, inflammasome, IL-1β, pyroptosis, acetylation, DNA methylation

## Abstract

Aberrant NLRP3 (NOD-, LRR-, and pyrin domain-containing protein 3) inflammasome activation in innate immune cells, triggered by diverse cellular danger signals, leads to the production of inflammatory cytokines (IL-1β and IL-18) and cell death by pyroptosis. These processes are involved in the pathogenesis of a wide range of diseases such as autoimmune, neurodegenerative, renal, metabolic, vascular diseases and cancer, and during physiological processes such as aging. Epigenetic dynamics mediated by changes in DNA methylation patterns, chromatin assembly and non-coding RNA expression are key regulators of the expression of inflammasome components and its further activation. Here, we review the role of the epigenome in the expression, assembly, and activation of the NLRP3 inflammasome, providing a critical overview of its involvement in the disease and discussing how targeting these mechanisms by epigenetic treatments could be a useful strategy for controlling NLRP3-related inflammatory diseases.

## 1. Introduction

Inflammation is a defense mechanism developed by our own immune system to fight infections, endogenous signals and tissue damage, and to restore cellular homeostasis. However, when the initial stimulus is too intense or long-lasting, it might lead to exacerbated damage that contributes to the pathophysiology of a wide range of diseases. Initially, the inflammatory response is triggered by cells of the innate immune system, such as macrophages and dendritic cells, which sense signals of damage, transfer that information to the inner cell, and respond by promoting the transcription of cytokines and antimicrobial peptides that are aimed at eliminating the initial damage. Inflammasomes are multiprotein complexes located within those innate immune cells that act as central nodules of the inflammatory response [1]. They can sense the danger signals, integrate them in the cell and respond by producing specific pro-inflammatory cytokines (IL-1β and IL-18) and a caspase-dependent form of cell death called pyroptosis. These complexes are not only restricted to immune cells but are also present in epithelial cells, mesenchymal stem cells, Langerhans islets and microglia, where they contribute to the progression of multiple inflammatory disorders such as atherosclerosis and Alzheimer’s disease [2,3,4].

The activation of inflammasomes begins when cells recognize pathogen-associated molecular patterns (PAMPs) or danger-associated molecular patterns (DAMPs) by pattern recognition receptors (PRRs). To date, and taking into account the cellular localization, two major families of PRRs have been reported. One of them is located in the plasma membrane and endosomes and is made up of Toll-like receptors (TLRs) and C-type lectin receptors (CLRs). A second family is located in the intracellular compartments and comprises RIG-I-like receptors (RLRs), the absent in melanoma 2 (AIM2)-like receptors (ALRs), and nucleotide-binding and oligomerization domain (NOD)-like receptors (NLRs) [5]. The NLR gene family shares a common structure, formed by an N-terminal domain, a central domain required for oligomerization (NOD), and a C-terminal domain of leucine-rich repeats (LRRs) that binds to ligands and sense signals. The NLR family is sub-divided into four groups, based on characteristics of the N-terminal domain. NLRA, NLRB, NLRC and NLRP have an acidic transactivation domain, a baculovirus IAP repeat domain (BIR), a caspase-recruitment and activation domain (CARD), and a pyrin domain (PYD), respectively. Some of these receptors trigger signaling cascades mediated by nuclear factor-κB (NF-κB), mitogen-activated protein kinases (MAPKs), activator protein 1 (AP1), and interferon regulatory factors (IRFs) whose function is to stimulate innate immunity. However, other receptors can recruit and activate the inflammatory caspase-1 forming complexes called inflammasomes.

The NLRP3 (NOD-, LRR-, and pyrin domain-containing protein 3) inflammasome is one of the most widely studied to date due to its ability to sense diverse pathogens and a large number of sterile signals such as crystals, ATP, aggregates such as β-amyloid, K^+^ efflux, and chemical sensitizers [6,7,8,9]. Upon activation, NLRP3 proteins oligomerize and recruit the adapter molecule apoptosis-associated speck-like protein (ASC, also known as PYCARD) that contains a CARD domain whose function is to activate pro-caspase-1 [10]. Once activated, caspase-1 promotes the cleavage of the pro-IL-1β and pro-IL-18 allowing the secretion of mature cytokines. However, caspase-1 also cleaves gasdemin D (GSDMD), a protein that oligomerizes and creates pores in the plasmatic membrane inducing a specific form of programmed cell death called pyroptosis [11]. The NLRP3-inflammasome is initially generated to restore cellular homeostasis and to avoid infections or situations in which damage may occur. Nevertheless, exacerbated NLRP3 inflammasome activation is involved in the pathogenesis of many diseases such as Alzheimer’s, rheumatoid arthritis, renal diseases and tumors, all of which are characterized by significant chronic inflammation [12,13,14]. It has been associated with the ability of NLRP3 inflammasome to sense and be activated by a plethora of DAMPs, including β-amyloid plaques, cholesterol and uric acid crystals, and oxidized low-density proteins.

Given its involvement in disease, many efforts have been directed towards discovering effective compounds or therapeutic strategies that can inhibit the NLRP3 inflammasome components or block its main final product. One of the first and most effective therapies has been the use of antibodies and molecules whose function is to block the IL-1β cytokine or its receptor. Some of them—Canakinumab, Anakinra and Rilonacept—have been approved by the US Food and Drug Administration (FDA) for the treatment of inflammatory diseases [15]. Further strategies have focused on targeting the inflammasome components to impair its assembly and further activation. The diarylsulfonylurea-containing compound, MCC950, is a specific inhibitor of NLRP3 inflammasome activation that abrogates ASC oligomerization in human macrophages and in in vivo models of multiple sclerosis and Parkinson´s disease [16,17]. MCC950 interacts with the NACHT domain of wild type NLRP3 but not with NLRP3 mutants associated with cryopyrin-associated periodic syndrome (CAPS), suggesting that there is a lack of effectiveness in that type of pathologies [18]. At the same time, Youm et al. [19] reported that the BHB (β-hydroxybutyrate) ketone metabolite can inhibit the K^+^ efflux and reduces the degree of oligomerization and ASC speck formation during canonical NLRP3 inflammasome activation in mouse models of familial cold auto-inflammatory syndrome (FCAS) and Muckle-Wells syndrome (MWS). Moreover, its therapeutic potential has been demonstrated in several pre-clinical models of NLRP3-mediated inflammatory diseases [20,21,22,23]. Additional inhibitors have been reported in recent years that target different sites of action through direct or indirect mechanisms. Some have shown their potential in in vivo models of disease. These include the NF-κB inhibitors (Parthenolide and Bay 11-7082), caspase-1 inhibitors (VX-740 and VX-765), the inhibitor of NLRP3-NEK7 (NIMA-related kinase 7) binding, oridonin, and the β-sulfonyl nitrile compound, OLT1177, which blocks the ATPase activity of NLRP3 and is currently the subject of a phase I clinical trial. [24,25,26].

Despite the great advances in this field, the lack of knowledge about the NLRP3 inflammasome regulatory mechanisms hinders research progress in the search for new strategies. The study of the epigenetic mechanisms involved in regulating the NLRP3 inflammasome components has given rise to a new way of understanding the underlying processes that regulate NLRP3 inflammasome activation and of identifying new therapeutic targets. Epigenetic regulation mechanisms, or heritable changes caused by external or internal environmental factors that modulate gene expression without altering DNA sequence, have been widely associated with the development, activation, and differentiation of immune cells [27,28,29]. These epigenetic modifications bestow upon cells the ability to adapt and respond to different stimuli according to the physiological or pathological conditions in which they are found [30,31].

Here, we will review the epigenetic mechanisms (DNA methylation, histone modifications and miRNAs) that are directly or indirectly involved in the expression of the components of NLRP3 inflammasome and the consequences of their modulation. Understanding these mechanisms highlights the potential value of epigenetic drugs for treating chronic inflammatory diseases in which NLRP3 inflammasome activation plays an essential role.

## 2. Activation of the NLRP3 Inflammasome: Two Steps with a Unique End

The expression of the components of the NLRP3 inflammasome, as well as its assembly and activation are processes that are strongly regulated by two sequential steps; a first step in which key components of the inflammasome are primed or transcribed, and a second in which a signal leads to the activation of NLRP3.

### 2.1. The First Step: Priming the NLRP3 Inflammasome

**Figure 1:**
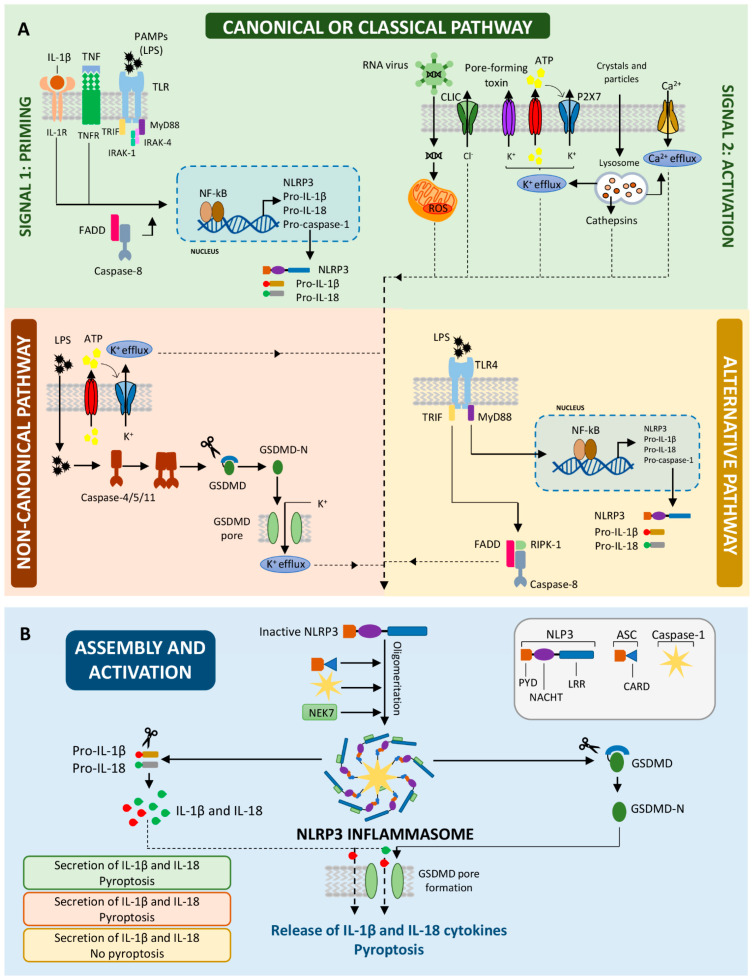
Pathways involved in NLRP3 inflammasome priming and activation. (**A**). Activation of the NLRP3 inflammasome requires an initial priming step aimed to upregulate the expression of inflammasome components. That first signal, or priming (left panel), is provided by PAMPs that induce NF-_k_B activation, thereby triggering the transcription of NLRP3, caspase-1, pro-IL-1β, and pro-IL-18. The FADD-caspase-8 complex is essential in this step since it allows NF-_k_B to be activated. After that, the second signal is accomplished by a wide range of stimuli, such as RNA viruses, pore-forming toxins, extracellular ATP, and crystals. These stimuli alter cellular homeostasis (changes in Ca^2+^, K^+^ and Cl^−^ flux, ROS production, mitochondrial dysfunction) triggering the assembly of NLRP3, ASC caspase-1 and NEK7 proteins, and establishing the activation of the NLRP3 inflammasome. Otherwise, a non-canonical pathway is activated when LPS from Gram-negative bacteria is internalized. Human caspase-4/5 or mouse caspase-11 can directly recognize the LPS and cut GSDMD, promoting the formation of pores in the plasma membrane. At the same time, these pores facilitate the egress of intracellular K^+^, thereby triggering the activation of the NLRP3 inflammasome. In monocytes, the recognition of LPS by TLR4 guides the activation of the alternative pathway in which priming is the only necessary step. The inflammasome is activated by the multiprotein complex FADD-RIPK1-caspase-8, independently of the K+ efflux, that leads to a gradual release of IL-1β and IL-18. (**B**). After assembly and activation of the NLRP3 inflammasome components by the different pathways, caspase-1 is activated inducing the cleavage of the immature forms of IL-1β and IL-18 cytokines and their further release to the extracellular medium. Moreover, active caspase-1 cleaves gasdermin D (GSDMD), which forms pores in the plasma membrane that give rise to cell death by a process named pyroptosis. Although this last process is not triggered by the alternative pathway.IL-1R: IL-1β receptor. TNFR: TNF receptor. PAMPs: pathogen-associated molecular patterns. FADD: FAS-associated death domain protein. PYD: pyrin domain. LRR: leucine-rich repeat. ASC: apoptosis-associated speck-like protein. NEK7: NIMA-related kinase 7.

Once that PAMPs or DAMPs are recognized by TLRs, myeloid differentiation factor 88 (MyD88) mediates the phosphorylation and degradation of IκB allowing the activation of NF-κB transcription factor [32]. As a consequence, NF-κB is translocated to the nucleus, where it promotes the transcription of NLRP3, caspase-1 and the non-active pro-forms of the IL-1β and IL-18 cytokines (pro-IL-1β and IL-18) (Figure 1A). It has been reported that FAS-associated death domain protein (FADD) and caspase-8 are also key regulators of NLRP3 expression during the priming step [33]. FADD is necessary to enable the maturation of caspase-8, which binds to IκB kinase, triggering the activation of the NF-κB pathway.

Otherwise, rapid NLRP3 activation might occur independently of the synthesis of new proteins. Simultaneous priming of bone marrow-derived macrophages (BMDMs) with LPS (lipopolysaccharide), with or without ATP, is enough to activate caspase-1, release pro-inflammatory cytokines and induce pyroptosis [34]. Signaling mediated by the TLR4/MyD88 pathway activates the IL-1 receptor-associated kinases, IRAK-1 and IRAK-4, allowing the assembly and activation of NLRP3 [35]. Thus, this mechanism licenses the cells to respond rapidly to pathogenic infections such as the caused by Listeria monocytogenes.

### 2.2. The Second Step: Activating the NLRP3 Inflammasome

Once all the components have been expressed in sufficient amounts, a second step is required for the oligomerization, assembly and activation of the inflammasome. This process begins with the activation of NLRP3 by a wide range of highly diverse molecules, including microbial stimuli such as nigericin or gramicidin, pore-forming toxins, endogenous signals such as urate crystals, extracellular ATP or heparan sulphate, and inorganic crystalline structures such as nanoparticles, silica, asbestos and necrotic cells [9,36,37,38,39] (Figure 1A). The direct interaction of NLRP3 with these molecules remains unconfirmed, but it is known that these stimuli can alter the cellular homeostasis by ionic and non-ionic mechanisms, allowing the NLRP3 inflammasome assembly and activation.

Alterations produced in the cells range from changes in the efflux of potassium, calcium and chloride ions to lysosomal destabilization, mitochondrial dysfunction, and production of reactive oxygen species (ROS) [40,41,42]. Perregaux et al. described for the first time that the maturation of IL-1β depends on the K^+^ efflux produced by ATP or nigericin stimulation [43]. Subsequent studies corroborated that K^+^ efflux is a necessary upstream activator of the NLRP3 inflammasome [8,44]. In response to microbial toxins, high concentrations of extracellular ATP trigger the activation of P2 × 7 receptor (P2X7R), an ATP-gated cation selective channel, which opens large nonselective pores in the membrane thereby facilitating access to NLRP3 agonist and decreasing intracellular K^+^. The resulting low levels of potassium cause the assembly of the NLRP3 inflammasome and thereby the maturation of IL-1β. Another proposed model involves lysosome destabilization produced by the engulfment of large molecules (crystals, β-amyloid) that damage the phagolysosome and release the lysosomal protease, cathepsin B, to the cytosol which goes on to activate NLRP3 [9,38]. The production of mitochondrial ROS promotes the binding of the thioredoxin-interacting protein (TXNIP) to NLRP3, accelerating its activation [45].

After these alterations activate NLRP3 in a not very well-defined way, the NLRP3, ASC and caspase-1 proteins are usually assembled in the trans-Golgi network [46]. To enable this, NLRP3 is composed of three main domains: an amino terminal pyrin domain (PYD), a NATCH core domain with ATPase activity essential for the oligomerization of the NLRP3 protein itself and for its activation, and an LRR domain that controls the activation of NLRP3 (Figure 1B). Upon activation, the LRR domain senses the stimulus, allowing the activation of the NATCH core domain and with it, the oligomerization of NLRP3, leaving the PYD domain exposed. The PYD domain interacts with its homonym domain in the ASC protein [47]. ASC, in turn, interacts with the effector molecule, caspase-1, through its CARD domain acting as a bridge between NLRP3 and caspase-1. For this to occur, several ASC dimers must oligomerize amongst themselves to form a large multiprotein complex, known as the ASC speck or ASC pyroptosome. These complexes are visible in most cells because of their great size. Only one speck is formed in each cell undergoing activation, for which reason it is considered a hallmark of inflammasome activation.

In recent years, new regulators of the assembly and activation of NLRP3 inflammasome have been identified. NEK7 is a serine-threonine involved in mitosis that interacts through its catalytic domain with the NOD and LRR domains of NLRP3 in a process dependent on ROS-induced NEK7 phosphorylation (Figure 1B) [48]. This molecule is essential not only for the activation of NLRP3 inflammasome but also for mitosis, two events which cannot occur simultaneously and in which the change may be modulated by NEK7.

However, not all cells require this two-step model to activate the NLRP3 inflammasome. Gaidt et al. [49] reported an alternative pathway for NLRP3 activation in human monocytes, which acts simultaneously with the priming step in the canonical pathway. In response to LPS, monocytes activate the TLR4-TRIF-RIPK1-FADD-CASP8 pathway, leading to activation of caspase-8 upstream of NLRP3, and, independently, to changes in K^+^ efflux, and to the formation of ASC specks (Figure 1A). This pathway allows the gradual release of IL-1β without trigger pyroptosis, suggesting that its role in cellular activation is more important than its effector functions that lead to the cell death. Conversely, it has been shown that the second signal alone (mediated by nigericin) is sufficient to induce cleavage of caspase-1 and the release of IL-18 by pyroptosis, but not IL-1β [50]. It is the result of the constitutive expression of pro-IL-18 and pro-caspase-1, while the pro-IL-1β transcription requires priming.

### 2.3. Non-Canonical NLRP3 Inflammasome Activation

Gram-negative bacteria can induce NLRP3 inflammasome activation by a caspase-1-independent pathway that is nevertheless mediated by capsase-11 (in mice) or caspase-4/5 (in humans) [51]. This is known as the non-canonical pathway, in contrast to the canonical pathway that is mediated by caspase-1 (Figure 1A). In this pathway, when LPS is present in large amounts or in outer membrane vesicles generated by Gram-negative bacteria, it can reach the cytosol of cells independently of the classical TLR4 pathway [52]. Alternatively, LPS binds to the high-mobility group box 1 (HMGB1) protein, which triggers its endocytosis [53]. Pro-caspase-11 directly or indirectly detects the presence of cytosolic LPS and is activated by the induction of the cleavage of the GSDMD that leads to the formation of plasma membrane pores [54]. Consequently, these pores facilitate the release of danger signals, such as the alarmins HMGB1 and IL-1α, and reduce intracellular K^+^ levels, which triggers the activation of the NLRP3 inflammasome, and in turn, the release of pro-inflammatory cytokines by mechanisms comparable to the canonical pathway. Thus, non-canonical inflammasome contributes mainly to the sepsis to fight against Gram-negative bacteria that escape the initial immune response [55]. Moreover, studies of Caspase 11^−/−^ mice models have shown its contribution to diverse lung and renal diseases induced by LPS, but also to acute gouty arthritis or dextran sulfate sodium (DSS)-induced colitis [56,57,58,59]. Other molecules, such as oxPAPC (oxidized 1-palmitoyl-2-arachidonoyl-sn-glycero-3-phosphocholine) or Leishmania lipophosphoglycan trigger caspase-11 activation, providing new situations in which the non-canonical pathway might be involved [60,61].

### 2.4. Release of Pro-Inflammatory Cytokines and Pyroptosis

Under NLRP3 inflammasome assembly and caspase-1 activation, the pro-forms of IL-1β and IL-18 are cleaved to yield their mature forms for subsequent release into the extracellular space (Figure 1B). IL-1β, one of the most potent pro-inflammatory cytokines, is able to recruit neutrophils to the damaged site, induce the production of other cytokines and chemokines, activate endothelial adhesion molecules, and stimulate the response of Th17 cells [62]. Similarly, IL-18 induces the secretion of IFN-γ by NK and T cells, and other cytokines such as GM-CSF, IL-8, IL-1β, and TNF-α to increase the activation and recruitment of neutrophils [63]. Additionally, prostaglandins, leukotrienes or high-mobility group protein 1 (HMGB1) molecules are also released during the cell death of monocytes/macrophages, enhancing the inflammatory response [64,65]. Therefore, NLRP3 inflammasome activation helps trigger and perpetuate the inflammatory response according to the type or length of the initial stimulus.

As a consequence of NLRP3 inflammasome activation, a form of programmed cell death known as pyroptosis is induced to maintain homeostasis and eliminate unnecessary cells (Figure 1B) [66]. Activation of caspase-1 removes the carboxyl end of the pore-forming protein, GSDMD, inducing its activation [11]. Cleavage of GSDMD forms pores in the plasma membrane, dissipating cellular ionic gradients and promoting the entry of water into cells, cell swelling and osmotic lysis. As a result, pro-inflammatory cytokines and inflammatory factors or alarmins are released into the extracellular space, where they contribute to the inflammatory response. Whether cell death induced by pyroptosis contributes to aggravate the inflammation or alleviates the damage is dependent on the context, and the strength and duration of the response.

## 3. Epigenetic Dynamics in the Expression, Assembly and Activation of the NLRP3 Inflammasome

There is considerable evidence to suggest that epigenetics is a hallmark of chronic inflammation in many diseases [67]. In recent years, considerable efforts have been made to identify the epigenetic mechanisms (DNA methylation, histone modifications and non-coding RNAs) that regulate the expression of the NLRP3 inflammasome components (“direct mechanisms”) or the key regulators involved in its assembly and activation (“indirect mechanisms”).

### 3.1. Direct Epigenetic Regulation of the NLRP3 Inflammasome Components

#### 3.1.1. DNA Methylation

**Figure 2:**
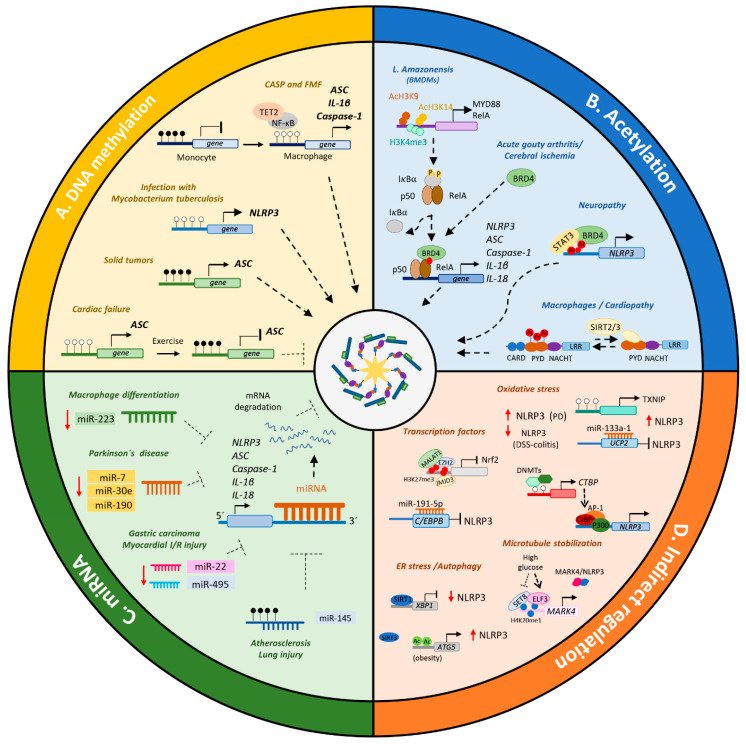
Direct and indirect mechanisms of epigenetic regulation modulating NLRP3 inflammasome activation. A variety of epigenetic mechanisms (DNA methylation, acetylation/deacetylation dynamics and miRNAs) are directly involved in the transcriptional regulation of inflammasome components. They are also indirectly involved through the modulation of factors and processes that participate in the assembly and activation of the NLRP3 inflammasome. (**A**). *DNA methylation*: During differentiation of monocytes to macrophages, regulatory regions of the *ASC*, *CASP1,* and *IL1B* genes are demethylated, thus enabling them to be expressed. In fact, NLRP3 is hypomethylated in infections and idiopathic syndromes to allow its transcription and further activation. ASC hypermethylation could condition the survival of tumor cells, and also contributes to alleviate cardiac failure, when exercise prevents its demethylation. (**B**). *Acetylation/deacetylation dynamics*: changes in the acetylation dynamics of genes involved in the NF-κB pathway lead to alterations in NLRP3, ASC, Caspase-1 and IL-1β transcription. During *Leishmania amazonensis* infection, impaired acetylation of H3K9 and H3K14 in MYD88 and RelA, blocks IκBα phosphorylation and NF-κB activation. Similarly, in acute gouty arthritis and cerebral ischemia, overexpression of BRD4 allows the expression of inflammasome components through activation of the NF-κB pathway. The recruitment of p-STAT3 and the acetylation of histones H3(K9) and H4 in the NLRP3 promoter region triggers its expression. Moreover, acetylation of the PYD domain of NLRP3 in macrophages is required for proper assembly with ASC. (**C**). *miRNAs:* downregulation of diverse miRNAs (miR-223, miR-7, miR-30e, miR-22, and miR-495) or hypermethylation of miR-145 during the development of the diseases leads to increased expression of all inflammasome components and further NLRP3 inflammasome activation. (**D**). *Indirect regulation:* epigenetic mechanisms involved in the expression of transcription factors (N2, C/EBPβ, CtBPs and XBP1), modulators of ROS production (TXNIP, and UCP2), genes encoding microtubule stabilization proteins, such as MARK4, and autophagy (ATG5) could facilitate changes in the expression of NLRP3 inflammasome components and stop the inflammasome activation.

Some of the first evidence that DNA methylation plays an important role in the regulation of NLRP3 inflammasome components was the presence of high DNA methylation levels in the promoter of ASC, CASP1 and IL1B genes in human monocytes [68] (Figure 2A). However, in monocyte-differentiated macrophages or monocytes activated with LPS and ATP/MSU, these genes were hypomethylated, allowing them to be expressed. This has been verified in monocytes from patients with CAPS and familial Mediterranean fever (FMF) syndromes in which a high level of demethylation of these NLRP3 inflammasome components is associated with the exacerbated expression of IL-1β. This loss of methylation is mediated by NF-kB and TET2 (Tet *methylcytosine dioxygenase 2*) demethylase, and treatment with anti-IL-1 drugs restores the methylation levels in CAPS patients at similar levels to those of healthy controls.

Several studies have demonstrated the essential role of DNA methylation in the regulation of the NLRP3 sensor. Wei et al. [69] reported that THP-1 cells infected with Mycobacterium tuberculosis and differentiated to macrophages had significantly lower levels of DNA methylation in the promoter of NLRP3 gene than in uninfected cells. This leads to a higher level of expression of NLRP3, inflammasome activation and release of IL-1β and IL-18 pro-inflammatory cytokines in response to infection. Idiopathic nephrotic syndrome (INS) is a chronic kidney disease whose pharmacological strategy is based on the administration of glucocorticoids to reduce inflammation, but around 20% of patients develop resistance to this treatment [70]. Analysis of the methylation pattern of NLRP3 in adult and pediatric patients with INS showed that patients with resistance to glucocorticoids had lower methylation levels than those that were sensitive to them, suggesting that NLRP3 inflammasome component expression and its activation contribute to steroid resistance [71].

Similarly, the ASC adaptor molecule is also modulated by changes in DNA methylation levels. The ASC gene is known to be hypermethylated in a range of tumor types, where it is associated with prognosis, although the results are mixed, varying with the type of tumor [72,73]. Hypermethylation of ASC is associated with lower survival rates and advanced disease stages in some tumors, such as non-small cell lung cancer, gastric cancer, renal cell carcinoma and lung adenocarcinoma. The loss of methylation and overexpression of ASC are associated with short-term survival, larger tumor size and greater tumor depth in glioblastoma, gastric cancer and oral squamous cell carcinoma patients [74,75]. Wu et al. [76] showed that low DNA methylation levels in the ASC gene are associated with a high level of expression of this protein in oral cavity squamous cell carcinoma tissues. Elevated mRNA levels of IL-1β, CASP-1, and NLRP3 were also detected, indicating that NLRP3 inflammasome has a role in that pathology. Overexpression of ASC improves cell migration and invasion, thereby favoring metastasis.

In addition to cancer, DNA methylation has a substantial influence on the regulation of ASC in heart failure (HF). The percentage of methylation in an intronic region of the ASC gene is inversely correlated with its expression and positively associated with a low risk of clinically adverse events [77]. Patients with HF who exercise exhibited higher ASC methylation levels, lower levels of ASC expression and impaired IL-1β release, all of which improve their aerobic capacity, quality of life and outcome [78].

#### 3.1.2. Histone and Non-Histone Proteins Acetylation and Epigenetic Readers

To date, many studies have shown the involvement of acetylation dynamics to be the main regulatory mechanism producing NLRP3 inflammasome activation (Figure 2B). Acetylation of specific residues in the histones not only allows the creation of an open chromatin structure accessible to transcriptional factors, but also facilitates the binding of epigenetic readers that can recruit all the transcriptional machinery needed to initiate activation of RNA polymerase II. During infection with the Leishmania amazonensis intracellular parasite, changes in the expression of pro-inflammatory genes (*MYD88* and *RELA*) in infected BMDMs are correlated with the loss of the histone activation marks, H3K9/K14 acetylation and trimethylation of H3K4 [79]. This leads to damaged NF-κB activation and ASC oligomerization, and thereby, a lesser degree of maturation of caspase-1 and IL-1β release. Therefore, L. amazonensis remodels the chromatin dynamics in macrophages to establish permissive conditions mediated by NF-κB and NLRP3 inflammasome downregulation, promoting its survival. In a murine model of painful neuropathy induced by bortezomib, the recruitment of p-STAT3 and the acetylation of histones H3(K9) and H4 in the NLRP3 promoter region triggers its expression and inflammasome activation [80]. Inhibition of NLRP3 expression attenuates the bortezomib-induced mechanical allodynia.

Additionally, the NLRP3 protein is modified by acetylation of lysine residues in the PYD domain in macrophages [81]. NLRP3 acetylation mediates the assembly with ASC and the activation of the inflammasome in response to LPS plus ATP. Conversely, the loss of the acetylation of NLRP3 mediated by the SIRT2 NAD^+^-dependent deacetylase, represses NLRP3 inflammasome activity. Aged SIRT2-deficient mice or those fed on a high-fat diet showed enhanced plasma glucose, insulin, and IL-18 levels consistent with increased activation of the NLRP3 inflammasome. Therefore, NLRP3 deacetylation by overexpression of SIRT2 reduces inflammasome activation in macrophages and prevents aging-associated chronic inflammation and insulin resistance. Likewise, SIRT3 deficiency in mice with diabetic cardiopathy increases NLRP3 expression, promoting the activation of caspase-1 and the maturation and cleavage of the pro-form of IL-1β [82].

BRD4 protein is a member of the bromodomain and extra-terminal domain (BET) family, that recognizes and binds to acetylated lysines in histones and non-histone proteins [83]. In several pathological mouse models in which NLRP3 inflammasome activation plays an essential role in the development and progression of the disease, blockage of BRD4 with pharmacological treatments (JQ1) or specific silencing reduces the production of inflammatory cytokines (IL-6, IL-1β, and IL-18) and pyroptosis [84,85,86,87]. Inhibition of BRD4 suppresses inflammasome activation by modulating NF-κB signaling, reducing the expression of all NLRP3 inflammasome components, and increasing the autophagy. However, the involvement of BRD4 in NLRP3 inflammasome activation in renal cell carcinoma has opposite effects [88]. Overexpression of BRD4 reduces caspase-1-dependent pyroptosis and enhances cell proliferation and epithelial mesenchymal transition progression. Thus, under tumoral conditions in which BET proteins are highly upregulated, blockage of BRD4 might be an exciting therapeutic strategy to induce the cell death by pyroptosis and avoid tumoral progression. Moreover, a recent pilot study suggests that histone acetylation and lysine-specific histone demethylase 2 (LSD2) are both involved in the NLRP3 inflammasome activation in clear-cell renal-cell carcinoma [89]. LSD2 is responsible for removing methyl groups from dimethylated and monomethylated H3K4 in NLRP3 gene, reducing its expression but not altering the caspase-1 and IL-1β levels.

#### 3.1.3. MicroRNAs

MicroRNAs (miRNAs) are epigenetic modulators that post-transcriptionally inhibit the expression of target genes. Several miRNAs are known to bind to the 3′-untranslated region of the NLRP3 gene, promoting its degradation (Figure 2C). Bauernfeind et al. [90]. have shown that during the differentiation of myeloid cells, low levels of miR-223 increase the extent of NLRP3 transcription, inflammasome activation and the release of pro-inflammatory cytokines in activated macrophages. In mouse models of Parkinson’s disease, the downregulation of miR-30e, miR-190, and miR-7 enables NLRP3 and ASC expression, the cleavage of caspase-1, the release of IL-1β and IL-18 cytokines, and the cell death by pyroptosis [91,92,93]. Overexpression of miR-495 is damaged in a mouse model of myocardial I/R injury, allowing the NLRP3, caspase-1, IL-18, and IL-1β expression [94]. It is well known that miRNA dysregulation during tumorigenesis favors the survival and proliferation of tumor cells and the development of metastasis. In Helicobacter pylori-induced gastric carcinoma patients, miR-22 downregulation increases NLRP3 expression, thereby facilitating progression of the disease [95].

In addition, miRNA expression can be modulated by epigenetic modifications, such as DNA methylation. In fact, in aortas from ApoE−/− mice fed on a western diet, miR-145 methylation increased the level of expression of NLRP3 and subsequently the release of soluble IL-1β, which contributes to plate formation [96]. Similar results were reported in an acute lung injury model with miR-495, where its hypermethylation promotes the expression of NLRP3 and other inflammasome components (ASC, caspase-1, IL-1β) [97].

### 3.2. Indirect Epigenetic Regulation of NLRP3 Inflammasome Components

In addition to the previously described mechanisms of epigenetic regulation that directly target the expression of different NLRP3 inflammasome components, epigenetic modifications in transcription factors, oxidative stress mediators, genes involved in autophagy, or microtubules stability are also involved in the inflammasome activation (Figure 2C).

Nuclear factor-erythroid 2-related factor 2 (Nrf2), a transcription factor associated with redox homeostasis, modulates the NLRP3 inflammasome-mediated inflammatory processes in a different way dependent on the cellular context. On one hand, it functions as a positive regulator of NLRP3 inflammasome activation [98]. But, on the other, it had been described that the presence of the H3K27me3 repressive mark in the NRF2 promoter, decreases its expression inducing ROS production and inflammasome activation in murine models of Parkinson´s and colitis [99,100]. Thus, Nrf2 is also known to act as a negative regulator of NLRP3 inflammasome activity. C/EBPβ is another transcriptional factor involved in NLRP3 transcription. In a mouse model of age-related macular degeneration induced by amyloid β, overexpression of miR-191-5p binds to C/EBPβ mRNA 3′-UTR, downregulating its expression [101]. Recently, Sun et al. [102] showed that the DNMT1 and DNMT3a expression levels are downregulated in biopsies of osteoarthritis patients, in which NLRP3 inflammasome plays a key role in the inflammatory response. CTBP1 and CTBP2 genes are consequently demethylated promoting their expression and the transcription of NLRP3 and subsequent inflammasome activation. It had been clearly stablished that the endoplasmic reticulum (ER) stress is involved in inflammatory processes by upregulating NLRP3 inflammasome [103]. SIRT1 regulates the transcriptional levels of XBP1, either by decreasing the acetylation levels of XBP1s or upregulating the miR-182 expression that binds to XBP1 to inhibit its expression and, therefore, the NLRP3 inflammasome activation and pyroptosis [104,105].

NLRP3 contains a highly conserved disulfide bond between the PYD domain and the nucleotide-binding site, suggesting that it is susceptible to oxidative stress [106]. Thioredoxin-interacting protein (TXNIP) acts as an endogenous inhibitor of thioredoxin (TRX), an antioxidant agent, increasing intracellular ROS levels and thereby the extent of transcription of the NLRP3, IL18, and IL1B genes [107]. Peripheral blood lymphocytes from coronary artery disease (CAD) patients show overexpression of TXNIP induced by the loss of DNA methylation levels in its 3′UTR regulatory region, promoting inflammasome activation [108]. When high levels of ROS are produced, the inner mitochondrial protein UCP2 (mitochondrial uncoupling protein 2) is upregulated to control the proton motive force in the mitochondrial matrix and electron transfer chain activity, which leads to a reduction in ROS levels and to the inhibition of the NLRP3 inflammasome [109]. Silencing of UCP2 by miR-133a-1 overexpression enhances the cleavage of caspase-1 and IL-1β in H_2_O_2_-treated THP-1 cells, suggesting that UCP2 negatively regulates NLRP3 inflammasome activation [110]. Moreover, it has recently been reported that SIRT1 silencing in murine models of demyelination increases the acetylation of PGC-1α (PPARγ co-activator-1α) attenuating mitochondrial dysfunction and inflammasome activation [111].

Autophagy is essential to control inflammasome activation and to avoid excessive inflammation by removing endogenous inflammasome activators, inflammasome components and cytokines [112]. In peripheral blood mononuclear cells (PBMCs) from obese humans, low levels of SIRT3 lead to the acetylation of ATG5 that impairs the formation of autophagosomes and increases ROS levels and NLRP3 inflammasome activation [113]. Finally, the stability of microtubules is one of the requirements to enable ASC to approach NLRP3 in order to facilitate inflammasome assembly [114]. It has been reported that microtubule affinity regulating kinase 4 (MARK4) binds to NLRP3 allowing its trafficking along microtubules and delivery to microtubule minus end at MOTC (microtubule-organizing center) [115]. In diabetic patients, high glucose levels mediate the NLRP3 inflammasome activation by upregulation of the MARK4 expression in endothelial cells [116]. This upregulation is mediated by E74-like ETS transcription factor 3 (ELF3) that binds to the lysine methyltransferase SET8, which catalyzes the monomethylation of H4K20 in the MARK4 promoter region, inhibiting its expression.

## 4. Epigenetic Targeting of the NLRP3 Inflammasome

As we previously reported, NLRP3 inflammasome activation initiates an inflammatory response that, when constant and persistent over time, produces a chronic inflammatory state that is difficult to reverse and that aggravates the outcome of many inflammation-related diseases. However, we must bear in mind that the NLRP3 inflammasome can be beneficial in specific conditions. The production of NLRP3 inflammasome-mediated inflammatory cytokines and pyroptotic cell death are essential for fighting numerous infections and cancer. Therefore, the NLRP3 inflammasome must be fine-tuned to maintain cellular homeostasis in each disease-specific context. In this scenario, the recent development of epigenetic drugs could be considered to provide a crucial therapeutic opportunity to modulate the expression of the NLRP3 inflammasome components, and the assembly and activation of the NLRP3 inflammasome. We will now review the in vitro and in vivo disease models in which targeting epigenetic mechanisms has proved effective at modulating the development of the disease (Table 1).

### 4.1. Metabolic Disorders

The NLRP3 inflammasome is an important point of connection between metabolism, immune function, and inflammation. Changes in metabolism generate metabolites or small molecules that trigger NLRP3 inflammasome activation in immune cells leading to a long-term chronic inflammatory state [117].

Acute gouty arthritis (AGA) is an auto-inflammatory disease characterized by the deposition of MSU crystals in tissue that activate the NLRP3 inflammasome [118]. Increased BRD4 expression in MSU-induced AGA rat models has revealed that treatment of these animals with JQ1 reduces neutrophil infiltration and attenuates joint swelling and synovial inflammation [84]. Butyrate, one of the main short-chain fatty acids (SCFAs) produced by the gut microbiota from dietary fiber that acts as a potent HDAC inhibitor, downregulates the transcription and release of IL-1β in PBMCs from patients with gout and healthy donors by blockading class I HDACs [119]. Similarly, low levels of romidepsin, a selective inhibitor of class I HDACs, impairs the transcription and release of IL-1β and other cytokines (IL-6, IL-8) in MSU-stimulated PBMCs from healthy donors [120]. That effect is mediated, at least in part, by the increased expression of the SOCS1 gene, a known negative regulator of inflammation that targets inflammatory molecules for proteasomal degradation. Other endogenous HDAC inhibitor, the ketone body β-hydroxybutyrate (BHB), also decreases the IL-1β release in MSU-activated macrophages and LPS plus ATP-stimulated neutrophils by inhibiting the priming and assembly steps [22]. In fact, in a MSU-induced gout model, rats fed on a ketogenic diet showed reduced IL-1β serum levels and intra-articular exudate and synovial inflammation were less severe.

Curcumin is a yellow pigment derived from the spice turmeric that has been approved by the FDA on account of its antioxidant and anti-inflammatory characteristics. Among its many biological activities, curcumin acts as an epigenetic modulator to regulate acetylation/deacetylation processes, DNA methylation and miRNA expression. Yin et al. [121] reported that curcumin inhibits IL-1β release in BMDMs, avoiding LPS priming and inflammasome activation by different mechanisms. Moreover, it prevents K^+^ efflux and blocks mitochondrial transport, thereby impairing NLRP3 assembly. Curcumin can also interfere with additional inflammasome modifiers such as autophagy, ROS production, and changes in α-tubulin acetylation.

### 4.2. Organ-Specific Inflammatory Diseases

It is clearly established that the NLRP3 inflammasome contributes to the pathophysiology of cardiac diseases including hypertension, myocardial I/R injury, or atherosclerosis [122,123,124]. Although BRD4 is overexpressed in cardiac hypertrophy and its specific silencing inhibits the phosphorylation levels of NF-κB, reduces IL-1β protein secretion, and prevents the development of fibrosis [125], a direct connection between BRD4 and the regulation of NLRP3 inflammasome has not yet been established. Epigenetic therapy with sirtuin-activating compounds, such as resveratrol, is a promising strategy for improving cardiac dysfunction [126]. In mouse models of myocardial infarction, treatment with resveratrol inhibits NLRP3 and caspase-1 (p20) expression, blocks the nuclear accumulation of NF-κB and thereby the expression of pro-inflammatory IL-1β, IL-6, and TNF-α cytokines into the infarction area [127].

Atherosclerosis is characterized by a chronic low-grade inflammation of the vessel wall driven by the recruitment of monocytes and macrophages to the atherosclerotic plaque. The levels of miR-145 expression are reduced by hypermethylation during the formation of the plaque and the consequent inflammation. Therefore, treatment with 5-aza-2′-deoxycytidine (DAC) restores the expression of miR-145, damaging the NLRP3 inflammasome activation in ApoE−/− mice [96].

Acute pancreatitis (AP) is a severe inflammation of the pancreas characterized by an intestinal barrier dysfunction, leading to bacterial translocation and the development of multiple infections [128]. Butyrate ameliorates the pancreatic damage and inflammation, reducing the extent of infiltration of neutrophils and macrophages into the pancreas, and promoting macrophage polarization to anti-inflammatory M2 macrophages [129]. That effect of butyrate is mediated by inhibition of the interaction between HDAC1 and AP1/STAT1 suppressing the NLRP3 inflammasome activity. Recently, Huang et al. [99] have reported that the demethylase JMJD3 is overexpressed in the DSS-induced colitis model upregulating the expression of Nrf2. Administration of GSK-J4, a specific inhibitor of JMJD3, reduces the production of IL-1β and with it the severity of the disease.

Deficiency of NLRP3, ASC or caspase-1 in mice with nephropathy induced by deposits of calcium oxalate in the renal tubules abrogates the inflammation and loss of renal function [130]. Administration of a diet containing a precursor of BHB, 1,3-butanediol, to an oxalate diet-induced nephrocalcinosis mouse model reduces renal inflammation, protecting the individual from chronic kidney damage [23]. High BHB levels reduce caspase-1 and IL-1β expression, decreasing the levels of inflammatory and pro-fibrotic markers and with it the creatinine levels. BHB induces the shift of infiltrating renal macrophages from a pro-inflammatory and pro-fibrotic phenotype to anti-inflammatory macrophages that restore the renal damage. In acute lung injury, aberrant DNA methylation of miR-495 is associated with elevated NLRP3 inflammasome activation [97]. In fact, restoration of miR-495 levels by an miR-495 mimic inhibits NLRP3, ASC, caspase-1, and IL-1β expression, alleviating the alveolar macrophage inflammation and pyroptosis induced by NLRP3 inflammasome activation. Osteoarthritis (OA) is characterized by damage or loss of the cartilage in joints. Treatment of a human osteoarthritic chondrocyte cell line with the hypomethylating agent, 5-azacytidine (AZA), induces demethylation of C-terminal-binding protein (CTBPs) genes, CTBP1 and CTBP2, increasing their expression [102]. As OA advances, aberrant expression of CtBPs facilitates the recruitment of AP-1 and the p300 HAT to the NLRP3 promoter, increasing its expression.

### 4.3. Neurological Disorders

Activation of the NLRP3 inflammasome by a wide range of exogenous or endogenous stimuli is associated with neuroinflammation, a feature of many neurological diseases [131]. Pharmacological inhibition of BRD4 by JQ1 impairs the NF-κB signaling, downregulating the expression of the pro-inflammatory cytokines, and reducing NLRP3 inflammasome activation and pyroptosis in cerebral ischemia-induced brain injuries [87]. Exogenous administration of several miRNAs (miR-30e, miR-190, miR-7) to Parkinson’s disease mice models inhibits NLRP3, ASC, and caspase-1 expression, impairing the release of IL-1β and IL-18, limiting pyroptosis and, consequently, neuroinflammation and neuronal damage [91,92,93].

BHB levels are lower in the red blood cells and brain parenchyma of Alzheimer’s patients, which suggests that BHB is important in the pathogenesis of disease [20]. Exogenous administration of BHB in murine models of Alzheimer´s disease reduces NLRP3 inflammasome activation (lower levels of ASC speck, caspase-1 activation, and IL-1β release), limiting plaque formation and microgliosis. The administration of a ketogenic diet to mice subjected to middle cerebral artery occlusion/reperfusion injury showed reduced ER stress and inhibition of the mitochondrial fission, which leads to decreased ROS production and NLRP3 inflammasome activation [21]. Cognitive impairment is also associated with increased neuroinflammation mediated by NLRP3 inflammasome activation and defective autophagy. In accordance with this, administration of the SAHA HDAC inhibitor to mice exposed to sevoflurane increases thereby downregulating the activation of the NLRP3 inflammasome and reducing the excessive inflammation [132].

### 4.4. Bacterial Infections

A considerable number of bacteria can elicit inflammasome activation in human cells of the mononuclear phagocyte system. Those affected are mediated by the direct binding of bacterial components such as LPS to inflammasome receptors, or by the recognition of these receptors of changes or alterations induced in the host cells by bacterial infections. These mechanisms are probably not mutually exclusive but act in combination to maintain the homeostasis against pathogenic infections.

Some bacteria, such as Mycobacterium tuberculosis (Mtb), can induce epigenetic changes in specific genes of host immune cells. Upon Mtb infection or treatment with a demethylating agent such as 5-aza-2′-deoxycytidine (DAC), the promoter region of the NLRP3 gene is demethylated, allowing it to be expressed and for inflammasome activation to occur [69]. Recently, it has been described that butyrate and propionate, can bind to the PYRIN domain of ASC and NLRPs, triggering the oligomerization of inflammasome complex and, thereby, the release of pro-inflammatory cytokines (IL-1β and IL-18) [133]. The administration of these SCFAs or high-fiber diets suppress Salmonella infection by recruiting inflammatory cells and activating the inflammasome. Nevertheless, inflammasome activation upon infection is not always satisfactory, and the response of host immune cells to bacteria components can cause an excessive response that damages tissue. For example, intestinal barrier dysfunction produces the bacterial translocation of microbial components such as LPS that goes on to activate the NLRP3 inflammasome, which contributes to the inflammation of the colon. Pre-treatment with the BET protein inhibitor, JQ1, protects the colon tight junction and inhibits NF-kB activation and NLRP3/ASC/caspase-1 expression in a mouse model of endotoxemia induced by LPS [85]. Likewise, the cleavage of gasdermins was also blocked by JQ1, regulating the cell death of colon cells.

### 4.5. Cancer

Inflammation and cancer are two related events, but the cellular and molecular mechanisms mediating this relationship are not fully understood. Inflammasome dysregulation has been most often associated with tumor prognosis, although there have been some controversial results reported. While some authors have shown a clear association between high levels of inflammasome activity and poor survival, for example in colorectal cancer and laryngeal squamous cell carcinoma [14,134], others have proposed NLRP3 inflammasome-dependent pyroptosis induction as a therapy for cases of acute myeloid leukemia or colorectal cancer [135].

DNA methylation is one of hallmarks of cancer, wherein it regulates the expression of oncogenes and tumor suppressor genes. Some studies have revealed the aberrant hypermethylation of ASC, suggesting that it could be considered a tumor suppressor gene [74,75]. Treatment with hypomethylating agents (i.e., DAC) restores ASC expression in ovarian, lung, breast and renal tumor cell lines, increasing the susceptibility of tumor cells to cell death by pyroptosis induced by doxorubicin [72]. Targeting acetylation/deacetylation dynamics have also been widely proposed for use in the treatment of cancer. SCFA, due to its activity as an HDAC inhibitor, has a potent inhibitory effect on the expression of all NLRP3 inflammasome components in addition to attenuating barrier dysfunction and ROS production, and activating the autophagy in Caco-2 tumor cells activated with LPS [136]. Blockage of BET proteins, specifically BRD4, is a promising therapy for the treatment of cancer due to the targeting of the protooncogene c-myc or the activation of NF-κB signaling, among others. Pharmacological inhibition of BRD4 with JQ1 inhibits cell proliferation and epithelial-mesenchymal transition in renal carcinoma cell lines and in vivo models in which a significant reduction of tumor size occurs [88]. JQ1 upregulates NLRP3 promoter activity, cleavage caspase-1, mature IL-1β and cleavage GSDMD, promoting cell-death by pyroptosis. Moreover, phosphorylation of NF-κB decreases significantly after JQ1 treatment, indicating that NLRP3-dependent pyroptosis is mediated by NF-κB inactivation. These results were previously reported in other studies in which NF-κB inactivation enhances the expression of NLRP3 [137]. Mitochondrial damage induced by the NLRP3 agonist triggers the production of NLRP3 inflammasome activators that are removed through p62-mediated mitophagy, which downregulates NLRP3 activation and restores cellular homeostasis [138]. In the absence of NF-κB activation, p62 expression is reduced, which promotes inflammation.

**Table 1:** Epigenetic treatments targeting the NLRP3 inflammasome activation.

Drug	Cell Line/In Vivo Model	Mechanisms of Action	Ref.
Hypomethylating agents			
AZA	Human chondrocytes osteoarthritis	Demethylation of *CtBP1* and *CtBP2* Increase of NLRP3 transcription	[102]
DAC	*M. tuberculosis*-infected THP-1 cells	NLRP3 demethylation and expression	[69]
	Atherosclerosis model	miR-145 demethylation and expression Downregulation of NLRP3 expression	[96]
	Renal cancer cell lines	ASC demethylation	[72]
Histone demethylases and deacetylases inhibitors			
GSK-J4	DSS-induced colitis model	Downregulation of Nrf2 expression Reduced NLRP3, Casp-1 and IL-1β expression	[99]
SCFA (Prop/But)	PBMCs from gout patients	IL-1β transcription downregulation	[119]
	Acute pancreatitis model	Inhibits HDAC1 and AP-1/STAT1 interaction decreasing NLRP3 activation	[129]
	*S. typhimurium* model	Increase inflammasome oligomerization	[133]
	Human Caco-2 cell line	Reduced ROS production.	[136]
BHB	Hyperoxaluria CKD model	Caspase-1 and IL-1β downregulation	[23]
	MSU-induced gout model	Inhibition of NF-kB phosphoirylation and NLRP3 priming and assembly.	[22]
	Alzheimer’s disease model	Low ASC speck and casp-1 activation	[20]
	Middle cerebral artery occlusion/reperfusion model	Decreased TXNIP and ROS production.	[21]
Romidepsin	Palmitic acid- and MSU-treated PBMCs from healthy donors	IL-1β transcription downregulation SOCS1 upregulation	[120]
Curcumin	LPS-stimulated BMDMs	NLRP3 priming and assembly inhibition	[121]
SAHA	Cognitive impairment model	NLRP3, caspase-1 and IL-1β downregulation	[132]
BET inhibitors			
JQ1	Acute gouty arthritis model	Low IL-1β levels and pyroptosis. Downregulation of NF-kB signaling.	[84]
	Acute colon injury model induced by LPS	Inhibit NF-kB activation and NLRP3/ASC/caspase-1 expression	[85]
	Cerebral ischaemia-induced brain injury	Inhibit NF-kB signaling and pro-inflammatory cytokines expression	[87]
	Renal carcinoma cell line	Upregulation of NLRP3 inflammasome activity and pyroptosis	[88]
Sirtuin activators			
Resveratrol	Myocardial infarction model	Inhibits NLRP3 and caspase-1 expression Blocks NF-κB nuclear accumulation	[127]
Exogenous miRNAs			
miR-30e,190,7	Parkinson’s disease model	Downregulation of inflammsome components	[91,92,93]
miR-495	Lung injury model	Downregulation of NLRP3 expression	[97]

AZA, azacytidine; DAC, decitabine; SCFA, short-chain fatty acids, BHB, beta hydroxybutyrate; MSU: monosodium urate crystals; PBMCs, peripheral blood mononuclear cells; BMDMs, bone marrow-derived macrophages; CKD, chronic kidney disease.

## 5. Conclusions

Activation of the NLRP3 inflammasome affects the development of a wide range of diseases but is also crucial to the host’s defense against bacterial infections and to the elimination of tumors. Thus, a finely tuned regulation of all the pathways that lead to the priming, assembly, and activation of NLRP3 is essential to ensure the modulation of the inflammation in the pathological or physiological contexts that may trigger the NLRP3 inflammasome. Considerable advances have been made in our understanding of the molecular mechanisms that lead inflammasome components to be expressed and of how different stimuli and processes can act together to facilitate the assembly of this macromolecular complex and, ultimately, the release of pro-inflammatory cytokines and cell death. Recent studies have begun to clarify how epigenetic mechanisms help to modulate the expression of NLRP3 inflammasome components and increase their stability, and thereby promote the assembly and activation of the inflammasome. Most of these studies have been carried out with epigenetic inhibitors using in vitro and in vivo mouse models of human diseases in order to evaluate the consequences of the inhibition for the inflammatory processes and the specific damage. It is now time to analyze how these epigenetic dynamics are initiated in diverse pathological situations and in response to different stimuli, and to establish whether they can integrate and respond to metabolic changes produced in the immune cells. Targeting DNA methylation and acetylation dynamics with demethylating agents and HDAC inhibitors led to a change in the treatment of many tumors, the majority of which are hematological cancers. Although these epigenetic drugs are not specific, they help to modulate the expression of proteins or molecules that are aberrantly expressed during oncogenesis. The best proof of this is that some of them have already been approved and are currently used in clinical practice. Although one step behind, epigenetic mechanisms are clearly involved in the regulation of pathways triggered during the inflammation. Before epigenetic treatments can be considered an effective therapy in this context, many aspects need to be addressed. First, we must ensure that they are safe and have reduced off-target effects, because most of them have a broad range of specificities. This is of considerable relevance because many chronic inflammatory diseases require long-term treatments. Moreover, robust studies with preclinical models are needed to demonstrate their potential to downregulate the heightened inflammatory response. Such an approach is essential if we are to determine whether epigenetic mechanisms are key to modulating and controlling the exacerbated NLRP3-mediated chronic inflammation that characterizes so many diseases.
